# Working Memory Training is Associated with Long Term Attainments in Math and Reading

**DOI:** 10.3389/fpsyg.2015.01711

**Published:** 2015-11-10

**Authors:** Stina Söderqvist, Sissela Bergman Nutley

**Affiliations:** Clinical Research, Pearson Clinical AssessmentStockholm, Sweden

**Keywords:** working memory training, academic attainment, cognitive training, cogmed, educational psychology

## Abstract

Training working memory (WM) using computerized programs has been shown to improve functions directly linked to WM such as following instructions and attention. These functions influence academic performance, which leads to the question of whether WM training can transfer to improved academic performance. We followed the academic performance of two age-matched groups during 2 years. As part of the curriculum in grade 4 (age 9–10), all students in one classroom (*n* = 20) completed Cogmed Working Memory Training (CWMT) whereas children in the other classroom (*n* = 22) received education as usual. Performance on nationally standardized tests in math and reading was used as outcome measures at baseline and two years later. At baseline both classes were normal/high performing according to national standards. At grade 6, reading had improved to a significantly greater extent for the training group compared to the control group (medium effect size, Cohen’s *d* = 0.66, *p* = 0.045). For math performance the same pattern was observed with a medium effect size (Cohen’s *d* = 0.58) reaching statistical trend levels (*p* = 0.091). Moreover, the academic attainments were found to correlate with the degree of improvements during training (*p* < 0.053). This is the first study of long-term (>1 year) effects of WM training on academic performance. We found performance on both reading and math to be positively impacted after completion of CWMT. Since there were no baseline differences between the groups, the results may reflect an influence on learning capacity, with improved WM leading to a boost in students’ capacity to learn. This study is also the first to investigate the effects of CWMT on academic performance in typical or high achieving students. The results suggest that WM training can help optimize the academic potential of high performers.

## Introduction

Working memory (WM) refers to the ability to keep information in mind and work with this information. WM has a limited capacity and can only hold a certain amount of information for a short period of time, in the order of seconds, before it decays. WM has been extensively studied and recognized as a fundamental cognitive function since it has been found to relate closely to other important cognitive functions such as reasoning ability, inhibitory control and attention (Engle et al., 1999; Conway et al., 2001, 2003; Kane et al., 2001, 2004). Importantly, WM capacity can influence the daily life of a child as it is associated with academic skills such as reading and mathematics both in typically developing children and children with ADHD (St Clair-Thompson and Gathercole, 2006; Sjöwall and Thorell, 2014). Moreover, WM capacity also has a predictive value for future academic performance. Sjöwall et al. (2015) studied children with ADHD longitudinally and found that WM capacity, together with other executive functions, measured at age 5–6 years, predicted academic achievements at 18 years of age. Such a relation is also evident in typically developing children, as WM has been found to predict future performance in both reading and mathematics (Bull and Scerif, 2001; Gathercole et al., 2003; Alloway and Alloway, 2010). Dumontheil and Klingberg (2012) found similar effects in a sample of typically developing children and adolescents (ages 6–16), with WM capacity predicting performance on an arithmetic tasks 2 years after baseline testing. In addition they found that WM related functional brain activity (measured as BOLD signal) in the intra-parietal sulcus predicted performance on the arithmetic task independently from behavioral measures. Taken together, these studies point to the importance of WM in academic attainment, not only for clinical groups but also for typically developing children and adolescents. The importance of WM capacity at an early age also suggests an important potential impact of interventions that aid WM development during childhood.

It has been shown that WM capacity can be improved through intensive computerized training, such as Cogmed WM Training (CWMT) and these effects have been shown to transfer to improvements in attention (Klingberg et al., 2005; Beck et al., 2010; Gropper et al., 2014). For example, Green et al. (2012) used an ecologically valid task to study effects on inattention in a randomized controlled trial of CWMT in children with ADHD. The outcome measure used (The Restricted Academic Setting Task) involved observers, who were blind to group allocation, recording symptoms of inattention in children while they were performing an academic task. They found significantly reduced symptoms of inattention (time spent off-task and time spent playing with objects) for the group who had trained CWMT compared to an active control group. Observations that WM training leads to improvements in both WM capacity and attention lead to the question of whether such improvements can also transfer to important every-day functioning such as learning in an academic setting.

Theoretically, there are several different routes by which improvements in WM capacity could lead to improvements in academic performance. One route could be by impacting the *learning* of academic skills. Within this reasoning, improving WM capacity and attention is assumed to help children pay more attention to what is being taught in class as well as stay more focused when carrying out their own school work, thereby aiding the learning process. In fact, one study has identified mind-wandering to mediate part of the association between WM capacity and reading comprehension (McVay and Kane, 2012), supporting this theory. An alternative route could be by the direct involvement of WM in many academic skills such as for example reading and mathematics. In this route an improved WM capacity would influence *performance* or application of already learned skills. These two routes are not exclusive of one another, rather it is likely that they are both important and even interact with each other in the learning process. Since WM training does not teach academic skills such as reading or mathematics, both routes of influence share the prerequisite that an individual is or has been within an environment that provides opportunities for learning these skills. In the case of the *learning* route it is also a prerequisite that sufficient time is allowed following training for the enhanced learning process to take place. This latter point speaks to the need for long-term follow up assessments within training studies, since effects on academic skills are only reasonable to expect directly after training if WM has been acting as a bottleneck for *performance* of the academic task.

Few studies have investigated this thus far with mixed results possibly reflecting the complexity of the many dimensions that underlie academic performance and learning. While some studies have reported improved academic performance after WM training (Dahlin, 2011, 2013; Loosli et al., 2012; Egeland et al., 2013; Holmes and Gathercole, 2013; Karbach et al., 2015), others have not demonstrated such effects (Gray et al., 2012; Dunning et al., 2013; Chacko et al., 2014; Ang et al., 2015; Redick et al., 2015). These studies have differed in the measures used to assess academic performance as well as the age groups and populations studied, which may explain some of the variation seen in the results. One other possible explanation for this could be the time allowed to pass between the completion of the intervention and the assessment of the learning outcome. Studies that have included a long term (>7 months) follow up measure have mostly, but not always (Dunning et al., 2013), reported effect sizes that have been substantially larger (Dahlin, 2011, 2013; Egeland et al., 2013; Holmes and Gathercole, 2013), than those merely assessing academic performance immediately or shortly after the completion of the intervention (Gray et al., 2012; Chacko et al., 2014). In a study by Dahlin (2011, 2013), children with special education needs have been observed to improve performance in reading and mathematics following CWMT. These improvements were found to remain significant at a 7-months follow-up. Similarly Egeland et al. (2013) concluded from a randomized, controlled trial that children with ADHD improved their reading fluency following training. This effect was found both when reading ability was measured directly after training completion and slightly increased 8 months later. Another aspect that differs between studies is the outcome measures used for assessment. For instance, while it may be valid to use standardized ability tests typically developed to screen for problem areas at baseline, they may not be suitable to assess specific academic progress or effects from interventions. In fact, none of studies that have used the most common standardized academic ability tests such as the Wechsler Individual Achievement Test or Wide Range Achievement Test have detected any effects after CWMT (Gray et al., 2012; Chacko et al., 2014; Ang et al., 2015). It may be that the effects are most evident when assessing learning on the actual school measures and subject areas currently being studied by the children and not with brief laboratory assessments providing a snapshot of specific fundamental academic skills. Although undoubtedly important, these skills might not reflect what a student has been learning post-intervention, nor provide a good reflection of more general scholastic achievement during the period for which a change is being assessed. Using actual school measures such as national academic tests or grades that are designed to measure specifically the skills covered in a specific period of education can therefore be assumed to offer a better measure of learning during that period. For instance, Holmes and Gathercole (2013) investigated a teacher led implementation of CWMT in an English school setting, training children who were underperforming academically. At the end of the school year, the students that had trained WM in the beginning of year 6 performed significantly better than students in the control group on the English and Math subtests of the national Standard Assessment Test (SAT). The training effects for the younger sample (grade five) were only evident in Math and were driven by an improvement in the training group as well as a decline in the control group.

While WM training interventions may benefit children with attention and WM impairments the most, it is also of interest to investigate whether transfer effects can be evident also in typical learners. Two studies have investigated this thus far, both using a WM training program that was primarily tapping verbal WM (Loosli et al., 2012; Karbach et al., 2015). Each of the two studies investigated training effects in elementary school children (aged 8–9) and found consistent results with improvements on WM and text reading using standardized curriculum based tests. The study by Karbach et al. (2015) included an active control group and demonstrated transfer to reading ability with a large effect size. Neither of the studies found effects on math performance. Taken together, these findings indicate that the impacting on learning through WM training is a complex matter which is likely a delicate interplay between the age at which the intervention is given, the tools used to assess a change in learning, the time allowed for learning to be impacted and the degree to which WM is the child’s limitation for academic growth. Academic benefits after training with Cogmed have been reported in children with either academic difficulties, a diagnosis of ADHD or with low WM, but it is yet to be determined whether benefits could be seen in typical learners as well. It is also unclear whether the potential effects could be sustained in the long term (>12 months).

In this study we investigated effects on reading and mathematics two years after CWMT had been completed by a school class of typical learners in Sweden. We hypothesize that students who completed WM training would show greater improvements on standardized school tests of mathematics and reading two years later when compared with an age matched control group. The second aim was to determine whether the magnitude of improvements in WM after training was related to the magnitude of improvements in scholastic achievement two years later. We hypothesized that training would be associated with a steeper development both in reading and math performance and that this development would, in both reading and math, be related to the magnitude in WM improvement during the intervention.

## Materials and Methods

### Participants and Procedure

Data was obtained from students in one classroom that had previously participated in an efficacy evaluation of a time reduced CWMT protocol, as well as a matched control classroom (C) from the same state school. The CWMT group (*n* = 20, 11 males, Mean age 9.85 years, *SD* = 0.32) had undergone training as part of their curriculum for 5 weeks, whereas the control group (*n* = 22, 8 males, Mean age 9.77 years, *SD* = 0.30) had continued with education as usual during the same period. The teachers from both classrooms agreed to provide individual results on academic performance on the Swedish National Standardized Testing in reading and math from the spring term of the school year prior to training (Year 3, *T* = 0) and to provide results on similar tests in both groups immediately prior to the intervention (autumn of Year 4, *T*1 = 6 months) and two years after the intervention (autumn of Year 6, *T*2 = 24 months post T1). According to school personnel no other interventions or substantial changes thought to intervene with their education occurred between T1 and T2. The math and natural science courses and the Swedish and social science courses were provided by the same two teachers across the classrooms, and some of their school work post intervention was performed with the original classroom groups mixed. Teachers reported that both classes had similar composition in terms of number of students with learning challenges (Dyslexia/dyscalculia: CWMT = 2, *C* = 2, ADHD/ADD: CWMT = 2, *C* = 1). There was no information linking the data to individuals. The teacher and school administrators gave written consent to provide the fully decoded academic data for the data-analysis and approved publication of the data. As the data used in this investigation was part of regular school activities and not linked to any personal data, informed consent was not necessary according to the guidelines of Swedish Central Ethical Review Board and thus not obtained. Both the reading and math outcome measures were collected in a group setting in the classrooms as part of the regular academic activities. The teachers thus gave the instructions and administered the tests according to regular test-taking procedures; in the classroom during silence with the teacher present during the whole administration. The students performed the tasks individually using paper and pencil. The WM intervention was the commercially available Cogmed^TM^ (Pearson Education, Inc.) which the teachers had extensive experience with (4 years at the time of training), and were well accustomed to implementing with training and coaching according to the Cogmed Coach Training standards. This included giving a start up session with background information on WM and CWMT, and to provide weekly feedback and support to motivate compliance to the intervention. The training took place during the school day in the computer room in the school and was performed in groups of around 10 students with two adults present at all times.

### WM Training Program

The intervention consisted of a time-reduced version of the traditional Cogmed RM (see for instance Egeland et al., 2013 for a detailed description of training tasks included). The traditional Cogmed RM consists of 12 verbal and visuo-spatial WM span exercises with a total of eight exercises to be completed in each training block. The time spent training in this study was reduced compared to traditional Cogmed RM by including fewer exercises per day (not fewer trials per exercise), resulting in four exercises to be completed in each training block. For each exercise, the task is to enter a sequence of presented stimuli in a precise order (either reproduced, backward or in a predefined order, e.g., ascending for numbers). The number of stimuli to-be-remembered presented for each trial adapts to the trainee’s performance on a trial-by-trial basis. This is to ensure that the load on WM is at each individual’s capacity limit, which has been shown to be a key component for increasing the capacity (Klingberg et al., 2005). There are seven visuo-spatial exercises and five verbal exercises. Every training block included exercises that taxed mainly the retention of information as well as exercises taxing retention and manipulation of information. The exercises included in each block rotated every few days to offer some variation for the trainees. Each completed training block ended with an option to play the reward game Roboracing, in which the trainees can compete with the robot mascot Stan (<1 min).

### Outcome Measures

The baseline measures (*T* = 0) on reading comprehension and math performance were the Swedish National Standardized Tests that are administered in the spring term of third grade. The sub-tests that were used for analyses were the reading comprehension of literature (Part B) and the mental arithmetic sub-test (Part D), as they were most comparable to the outcome measures used to assess the effect of WM training. The outcome measures used in this study were the individual students’ test performances on two age appropriate tests of reading and math given at Year four (T1) and six (T2). The reading consisted of the dimensions of reading rate, reading comprehension and spelling from the Diagnostic Reading- and Writing Test (DLS) (Järpsten and Taube, 2010). The math outcome used was the Adler Mathematical Screening test (Adler Matematik Screening, ©Adler, B., 2000) where the students are asked to solve a set of math problems using mixed operations in five min. These scores were standardized across the two groups for each time-point separately.

### WM Tests

The untrained measures of improvements in WM used were the three tests included in the Cogmed Progress Indicator (CPI) that appeared at baseline and then every five days throughout the training period. A version of the “Odd One Out” from the Automated Working Memory Assessment (Alloway, 2007) was used to measure visuo-spatial WM, which includes presentation of three figures and requires identification of the one figure that differs from the other two presented. Several sets of figures are then presented (number of sets equals difficulty level) and after presentation, the participant is asked to indicate the location of the odd figures in the order in which they were presented. The test ends when two trials on the same level are failed and the score achieved is the highest level where at least one trial was answered correctly, subtracting 0.3 for each incorrect trial on that level and 0.15 for each incorrect response on levels below that level. To assess the ability to follow instructions (FI), a digital version of the paper- and pencil version of such a task developed by Gathercole et al. (2008) was used. This test was administered and scored according to the same rule as the visuo-spatial WM task. A speeded mental arithmetic (SMA) task is also included in the CPI where as many math problems as possible should be solved during one min. The score is calculated as the number of correct trials subtracting the number of incorrect trials multiplied by a factor of 0.33. Improvement on all three CPI tests was calculated as the difference between the highest performance during the training period on each of the tests and the baseline score (highest performance on the first and second training instance) for the respective test. We also used measurements of improvements on training tasks, one verbal and one visuo-spatial. The verbal WM task (Input Module) consists of a panel with digits shown on the screen while a sequence of numbers are presented verbally. The response is then made by clicking on the digits in the reverse order from which they were presented. The visuo-spatial WM task (Visual Data Link) consists of a four-by-four grid of lamps that light up sequentially and the task is to remember the location and order of the sequence as it was presented. These two training tasks were selected for analyses to include both verbal and visuo-spatial domains and as they are the most frequently occurring during training. Improvements on the two training tasks were calculated by subtracting the baseline score on each task from the maximum score on the same task. Baseline score was calculated by averaging the level of the three most difficult correct trials on day one for that particular task. Maximum score was calculated by averaging the level of the three most difficult correct trials on that particular task throughout the whole training.

### Statistical Analysis

The statistical analyses were performed in R (R Core Team, 2014). Pearson’s *t*-tests were used to assess difference between the groups in baseline performance (at T0 and T1) and difference in age. Pearson chi-square test was used to assess difference in gender between the two groups. Reading and math scores were standardized across the groups for each time point separately. This was necessary since the assessments differed between the three time points, as they were age-appropriate academic measures that need to take into account the progress that has occurred during the two years between measuring points. The statistical tests investigate the relative change in distribution of individuals’ scores between the training and control conditions over time. The hypotheses were tested using linear regression models. To analyze the first hypothesis that WM training would be associated with greater improvement in reading, we performed linear regression analyses where the standardized score on the DLS (for grade six) at T2 was entered as a dependent variable and standardized score on DLS (for grade four) at T1 and training group (as a factor) were entered as independent variables. Similarly, to analyze the effect of WM training on improvements in mathematics we performed linear regression analyses with standardized scores on the Adler Mathematical Screening Test at T2 as dependent variable, and performance on the test at T1 and training group (as factor) as independent variables. To test the second hypothesis that the magnitude of improvements in WM after training was related to the magnitude of improvements in scholastic achievement two years later, we repeated the analyses for reading and mathematics independently, but now replacing the training factor with the different measures of training improvements (WM improvement, FI Improvement, Math Improvement, and improvement on the verbal and visuo-spatial training tasks). Each training improvement was entered as a continuous independent variable and a separate analysis was done for each measure of improvement. Cohen’s effect sizes were calculated to estimate the standardized size of the effect on the outcome measures, comparing average development for the two groups [(Δ*M*_1_*–*Δ*M*_2_*)/*Δ*SD*_pooled_].

## Results

The training statistics showed that the average active time for the training group was 22.77 min per training block (*SD* = 2.97) and on average 3.25 (*SD* = 1.72) training blocks were completed per week. All of the 20 students in the training group completed at least 20 training blocks (average blocks = 24.25, *SD* = 1.86) and were thus included in the analyses. The two groups did not differ in terms of age as tested with a Pearson’s *t*-test (*T* = 1.01, *p* = 0.31) or sex as tested with a Pearson’s chi-square test [χ^2^(1) = 0.37, *p* = 0.54]. For the outcome measures of reading and math, the two groups did not differ at either of the two baseline measures, as tested with Pearson’s *t*-tests (Reading T0: *T* = 0.74, *p* = 0.46, Math Year 3: *T* = 0.93, *p* = 0.36, Reading T1: *T* = 0.33, *p* = 0.74, Math T1: *T* = 0.63, *p* = 0.53). Furthermore, the groups did not differ in the development of neither reading nor math from T0 to T1, as assessed using linear regression analyses with reading or math performance at T1 as dependent variable and controlling for reading or math at T0 together with training group (as factor) as independent variables (training group: *p* = 0.92 for reading and *p* = 0.52 for mathematics). Baseline measures on the national standardized test at T0 indicate that the students overall are to be considered typical learners/high performers with 100% of the students reaching target level of attainment in reading (compared to 95.3% nationally), and 84.1% of the students reaching target level of attainment in math (compared to 86.3% nationally) (see **Table 1** for standardized scores for each time point). The effect size for the difference between the groups in the development on reading was 0.66 SD. The effect size for development on math performance between the groups was 0.58 SD.

**Table 1:** Mean standardized scores on reading and math performance (with standard deviations in brackets) for the training and control groups are summarized below.

	Reading		Math	
	Training	Control	Training	Control
T0	-0.13 (1.09)	0.11 (0.93)	-0.16 (1.17)	0.14 (0.82)
T1	-0.05 (1.14)	0.05 (0.87)	-0.10 (0.86)	0.09 (1.12)
T2	0.19 (1.24)	-0.22 (0.69)	0.12 (0.95)	-0.11 (1.1)

*The scores have been standardized for each time point separately and a negative development is thus only in contrast to the other condition.*

For reading, the training condition (entered as a factor) significantly explained variance in the outcome two years later (T2), while controlling for test performance at baseline (T1) (see **Table 2** for regression coefficients). For math performance, the training condition (entered as a factor) trended toward explaining variance in the outcome measure significantly, while controlling for baseline performance.

**Table 2:** Regression coefficients and *p*-values for the models describing the relation between the outcomes of math performance or reading two years following training and the training condition or improvements on the training outcomes controlling for T1 performance.

	Dependent variable: math T2				Dependent variable: reading T2			
Independent variables	Estimate (*B*)	*SE*	*T-*score	*p-*value	Estimate (*B*)	*SE*	*T-*score	*p-*value
Outcome at T1	0.76	0.11	6.90	<0.001	0.78	0.096	8.14	<0.001
Training group	0.37	0.22	1.73	**0.091**	0.40	0.19	2.07	**0.045**
	*Adjusted R*^2^ = 0.53, *p* < 0.001				*Adjusted R*^2^ = 0.63, *p* < 0.001			
Outcome at T1	1.02	0.14	7.37	<0.001	0.82	0.10	8.04	<0.001
WM Improvement	0.98	0.35	2.78	**0.0081**	0.21	0.25	0.84	0.41
	*Adjusted R*^2^ *=* 0.58, *p* < 0.001				*Adjusted R*^2^ = 0.61, *p* < 0.001			
Outcome at T1	0.91	0.15	5.99	<0.001	0.81	0.10	8.10	<0.001
FI Improvement	1.00	0.56	1.77	**0.085**	0.42	0.37	1.15	0.26
	*Adjusted R*^2^ *=* 0.53, *p* < 0.001				*Adjusted R*^2^ = 0.61, *p* < 0.001			
Outcome at T1	0.98	0.15	6.72	<0.001	0.81	0.10	8.08	<0.001
SMA Improvement	0.13	0.085	1.57	0.12	0.067	0.057	1.19	0.24
	*Adjusted R*^2^ *=* 0.53, *p* < 0.001				*Adjusted R*^2^ = 0.61, *p* < 0.001			
Outcome at T1	0.98	0.15	6.67	<0.001	0.77	0.097	7.92	<0.001
Training task imp. (Verb)	0.60	0.37	1.59	0.12	0.55	0.25	2.23	**0.031**
	*Adjusted R*^2^ = 0.53, *p* < 0.001				*Adjusted R*^2^ = 0.64, *p* < 0.001			
Outcome at T1	1.02	0.14	7.04	<0.001	0.81	0.099	8.15	<0.001
Training task imp. (VS)	0.65	0.32	2.00	**0.052**	0.30	0.22	1.38	0.17
	*Adjusted R*^2^ = 0.54, *p* < 0.001				*Adjusted R*^2^ = 0.62, *p* < 0.001			

*Predictors explaining variance in the outcome at trend or significant levels are in bold. Explanations of the independent variables: outcome at *T*1 = referring to the same measure (reading or math) as the dependent variable but measured pre-training (grade 4), Training group = training or control group coded as a factor, WM Improvement = Improvement on the untrained working memory (WM) test from the CPI, FI Improvement = Improvement on the untrained following instructions test from the CPI, SMA Improvement = Improvement on the speeded arithmetic test from CPI, Training task imp. (Verb) = Improvement on the verbal training task, and Training task imp. (VS) = Improvement on the visuo-spatial training task.*

The analyses to assess whether the magnitude of improvements in scholastic outcomes were related to the amount of WM improvement observed during the intervention demonstrated that the variance in reading improvement was significantly explained by the magnitude of the improvement seen on a verbal WM task during the intervention (Input module). For math development, the magnitude of improvements on the WM test from the CPI significantly explained variance in the math performance two years later while controlling for math performance at baseline (see **Table 2** for regression coefficients). This pattern was also evident on trend level for the Following Instructions test from CPI (*p* = 0.085) and a visuo-spatial WM task (Visual Data Link) from the training (*p* = 0.052).

## Discussion

Here we report on findings from a controlled WM training study assessing long-term effects on academic attainment. The results suggest that CWMT can affect learning outcomes as evident in reading and math performance two years following training. This is, to our knowledge, the first study to investigate ecologically valid transfer improvements following CWMT during a longer time period (>12 months). The fact that the training took place in a classroom setting with excellent compliance and with minimal involvement from the researchers, suggest both high efficacy and effectiveness of CWMT. Furthermore, these results demonstrate improvements in academic performance in a sample of “typical learners”, suggesting that benefits of CWMT are not limited to individuals with WM deficits. The version of CWMT used in this study was an altered version compared to the original researched paradigm and the dose was reduced to nearly half of the regular protocol. This suggests that benefits are evident also when using shorter training protocols, a finding that may be impactful for trainees that are unable (due to fatigue or practical reasons) to go through the rather demanding 40–50 min/day protocol originally developed (Klingberg et al., 2002, 2005).

Previous studies investigating transfer to academic achievement following CWMT have reported mixed results with some finding positive impact on reading (Dahlin, 2011; Egeland et al., 2013; Holmes and Gathercole, 2013) and math (Dahlin, 2013; Holmes and Gathercole, 2013; Bergman-Nutley and Klingberg, 2014), while others have not found statistically significant improvements on academic performance following CWMT (Holmes et al., 2009; Dunning et al., 2013; Chacko et al., 2014; Ang et al., 2015). In order to bring the scientific field forward it is important to try to understand which factors are underlying these differences in results.

This study investigated the academic progress in children, two years after completing a time reduced version of CWMT, compared with children in an age-matched class with a similar composition of students in terms of sex and share of children with academic or behavioral difficulties. While the results indicate that the group that underwent CWMT improved more on reading and math performance over time, there were also individual differences in the amount of benefits observed within the trained group. These differences were also reflected in the academic outcomes. This observed linear relation between improvements on the training tasks and the academic attainments supports an increased WM capacity as a likely explanation to the enhanced academic attainment in the training group. Previous studies have identified improvement on the trained tasks as an important predictor of amount of transfer seen to other tests (Jaeggi et al., 2011; Söderqvist et al., 2012; Bergman-Nutley and Klingberg, 2014; Karbach et al., 2015). What is particularly interesting in this study is that there seems to be differential domain specific relations between improvements on verbal and visuo-spatial training tasks, and the outcomes of reading and math performance, respectively. The improvements on the verbal WM training task were linearly related to the improvements in reading, while the improvements on the visuo-spatial grid task were linearly associated with the math attainments two years later. This is perhaps not surprising as the type of arithmetic test used here has been shown to correlate highly with visuo-spatial WM capacity in particular (Raghubar et al., 2010). Furthermore, activity recorded during this type of visuo-spatial WM task in the intra parietal sulcus (an area in the brain associated with numeracy and WM capacity), has been shown to predict performance on an arithmetic task two years later (Dumontheil and Klingberg, 2012). The test assessing reading included reading rate, reading comprehension and spelling, aspects that have been shown to correlate with primarily verbal WM (Seigneuric et al., 2000; Pham and Hasson, 2014). This is also consistent with the studies training primarily verbal WM demonstrating transfer to the reading test specifically and not the math outcome in typical learners (Loosli et al., 2012; Karbach et al., 2015).

The fact that no relation was detected between the amount of improvement on the speeded math task (part of the CPI) during training and the improvement on the math outcome at the follow up, might indicate that the mechanism leading to improvements over time are acting through the improved *learning* route in this case rather than directly affecting *performance.* Again, these routes are not exclusive from one another but this finding points to, at least in part, separate mechanisms that affect learning of math over the long term that may be more closely linked to utilizing a better functioning WM during formal instruction rather than an improved ability to apply current information during performance of a math task. The primary route of action may be different for trainees with an impaired WM capacity. In such cases the WM deficit may be directly limiting their ability to apply current knowledge and perform tasks to their full potential, as seen in the large-scale study by Bergman-Nutley and Klingberg (2014) where WM related improvements were seen on the same math task immediately after training. However, this is for future studies to determine.

The specificity of the relations between the training tasks and the outcomes suggests that WM tasks used in training may lead to differential effects depending on the exact processes tapped by the task. This could also imply that differential patterns of improvements between individuals (and thus also studies) in scholastic achievement could depend on whether verbal or visuo-spatial WM has been particularly impaired and/or particularly improved with training. This points to the importance of being able to predict for whom *which* type of training would be most beneficial and highlights the limitations of the one-size fits all paradigms currently being used. In order to improve our ability to accurately predict training outcomes, we must raise the standards of cognitive training studies and aspire to design and conduct studies that are large scale (properly powered), randomized, blinded, controlled, and that are carried out with high quality implementation, long term follow up assessments using relevant and sensitive outcome measures. This was not that study. This study was not randomized and only had a passive control condition, small sample size, and lacked cognitive assessments in the control group. However, active and blinded control conditions are employed primarily to control for placebo or Hawthorne effects (McCarney et al., 2007) and since this training was implemented entirely by the school teachers as part of their regular classroom activities, these types effects can be assumed to be small or non-existent. Furthermore, expectancy effects influencing performance on the outcome measures would also not be anticipated for either group, as they too were part of their regular school activities, differed substantially from WM tasks, and no information was given to the students that their math and reading scores would be analyzed in light of the WM training that one of the groups went through two years prior to the test. The small sample size limits the conclusions to be drawn from non-significant findings and warrants caution in the generalizability of the findings that should clearly be further explored, however, since the results are in line with part of the previously reported findings in typical learners (Loosli et al., 2012; Karbach et al., 2015) and in different populations (Dahlin, 2011, 2013; Egeland et al., 2013; Holmes and Gathercole, 2013), the conclusions are not unexpected.

A possible alternate explanation to these findings is that the students in the classroom training CWMT had higher quality instruction, or other uncontrolled benefits, and were more motivated in their school work in general, however, the similar baseline performance at T0 and T1 suggest otherwise. Furthermore, both classrooms had the same teachers in the courses in which reading and math proficiency would be expected to be improved the most. The linear relation between the training improvements and the improved outcomes further suggest that the CWMT at least played a role in this difference in improvement between the classes. Another possible alternate explanation to these findings could be that the results are due to fluctuation in performance that occur by chance (e.g., regression to the mean). However, such effects would have been expected to be visible already between T0 and T1, which was not the case since there were no significant difference between the two groups in neither reading nor math development during this pre-training time period. Therefore these results illustrate that CWMT, as implemented by a regular school as part of their curriculum, can contribute to increased academic attainment in typical learners that will be evident over time. The practical implications for these results highlight a more general role for cognitive functioning as an important aspect of education in order to ensure that students’ cognitive capacities are taken into consideration in the classroom in order to enable students’ full learning potential. More specifically, this could open up for a discussion of including WM training in the regular curriculum in schools. As some studies have demonstrated that effects are most beneficial for student’s with the lowest baseline performance (compensation effects; Dahlin, 2011; Karbach et al., 2015), implementing interventions for all students may consequently benefit the entire group by raising the lowest ability level in the classroom, thus evening out differences within a group of students. Including all students in these types of interventions may also help prevent further stigmatization around children with attention or WM difficulties.

Future studies should aspire to determine the predictors of responsiveness to WM training and assess factors such as age, baseline performance of both cognitive abilities and academic proficiency in order to better understand the conditions under which optimal training effects can be achieved. Unfortunately, these types of studies are expensive and cumbersome to undertake and progress is slow. In the meantime, the current state is that we know with certainty that WM capacity is key to controlled attention, problem solving and is essential in performing mental arithmetic and to understand communication (written and verbal), all crucial factors for scholastic performance. We also know that WM capacity can be improved with training. It is for future studies to explore with more certainty how these two relate.

## Conclusion

This study demonstrated effects on academic attainment, two years following CWMT as implemented entirely by teachers in a classroom with typical learners. The trained group showed a steeper development in reading and math compared with a matched control group (with medium effect sizes) at the two-year follow-up assessment. Furthermore, the developments in both math and reading for the trained group were linearly related to the amount of improvements seen on the WM tasks. These findings suggest that CWMT could help accelerate learning in typical learners with as little as 20 min of training/session for 25 sessions.

## Conflict of Interest Statement

At time of submission Stina Söderqvist and Sissela Bergman Nutley are both employees of Pearson Clinical Assessment, the distributors of Cogmed Working Memory Training which is used in this study.
